# First report of cultivated Cretan mountain tea (*Sideritis syriaca*) as a host of *Meloidogyne hapla* and *M. javanica* in Crete, with some additional records on the occurrence of *Meloidogyne* species in Greece

**DOI:** 10.21307/jofnem-2019-010

**Published:** 2019-04-16

**Authors:** Emmanuel A. Tzortzakakis, Carolina Cantalapiedra-Navarrete, Antonio Archidona-Yuste, Maria Kormpi, Juan E. Palomares-Rius, Pablo Castillo

**Affiliations:** 1 Department of Viticulture, Vegetable Crops, Floriculture and Plant Protection, Institute of Olive Tree, Subtropical Crops and Viticulture, N.AG.RE.F., Hellenic Agricultural Organization-DEMETER, Heraklion, Greece; 2 Institute for Sustainable Agriculture (IAS), CSIC, Avenida Menéndez Pidal s/n, 14004, Córdoba, Spain; 3 Benaki Phytopathological Institute, Athens, Greece

**Keywords:** *Meloidogyne hispanica*, *Meloidogyne incognita*, Corn, Soybean, Aloe

## Abstract

Cultivated Cretan mountain tea or Malotira (*Sideritis syriaca* L.) was found to be infected by *Meloidogyne hapla* and *Meloidogyne javanica* in the island of Crete. The authors provide the first molecular characterization of *M. hapla* in Greece and the first report of Cretan mountain tea or Malotira as a host of *Meloidogyne* species worldwide. In addition, *Meloidogyne hispanica* was found infecting aloe (Andros island) and corn (Drama, North Greece) consisting the first reports of natural infection of these plants by *M. hispanica* in Europe. Furthermore, infection of corn by *M. incognita* and soybean by *M. javanica* (Drama, North Greece) are reported for the first time in Greece. Integrative taxonomical approach based on perineal pattern and EP/st ratio, as well as the region of the mitochondrial genome between the cytochrome oxidase subunit II (*coxII*) and 16S rRNA mitochondrial DNA (mtDNA) genes was used to differentiate *Meloidogyne* species.

Root-knot nematodes (RKNs), *Meloidogyne* spp., are recognized worldwide as the most economically important nematodes in agriculture, with a wide host range, and common in the Mediterranean area (Lamberti, 1981; Karssen and Moens, 2006). Knowledge of their occurrence in agricultural land is of vital importance for designing control measures. However, *Meloidogyne* spp. identification is complex, difficult and time-consuming, even for experts. In Greece, RKNs have been found in several locations and their identification was based on morphologic and morphometric characters and/or differential host tests until the middle of 1990s (Tzortzakakis et al., 2011). Within the last 20 years, the three major RKN species *M. javanica* (Treub, 1885; Chitwood, 1949); *M. incognita* (Kofoid and White, 1919; Chitwood, 1949); *M. arenaria* (Neal, 1889; Chitwood, 1949); and also *M. ethiopica* (Whitehead, 1968) and *M. hispanica* (Hirschmann, 1986) have been identified with molecular and/or biochemical markers (Tzortzakakis et al., 2011, 2014; Conceicao et al., 2012). In a recent publication, it was indicated that all of the reports of *M. ethiopica* from Europe, including the one from Greece, should actually refer to the species, *M. luci* (Carneiro et al., 2014; Geric Stare et al., 2017).

In spring–summer 2017, root and soil samples from five fields with RKN infestation from Heraklion Province, Island of Crete, Island of Andros and Drama province, North Greece were examined to identify the RKN species and to report the results herein. Egg masses were picked and put in water to release second-stage juveniles and males, whereas additional single egg masses were used to inoculate potted tomatoes (*Solanum lycopersicum* L.). In two cases of the corn (*Zea mays* L.) samples where mature egg masses were not found in galled roots, the original soil from the rhizosphere was used to fill pots (1,000 cm^3^) to which tomatoes were planted. After a 50 day growing period in a growth room, at 23–26 °C and 16 hr photoperiod, females, egg masses, second-stage juveniles and males were isolated from the tomato roots. All of the nematode stages isolated from the infected plants and the potted tomatoes were used for nematode identification.

Perineal patterns of mature females were prepared according to standard procedures (Hartman and Sasser, 1985). Briefly, root tissues were teased apart with forceps and half spear to remove adult females. The perineal pattern was trimmed and transferred to a drop of glycerin. In each sample, perineal patterns and the distance from excretory pore to the anterior end/stylet length ratio (EP/st) character were established for species identification in more than 10 mature female specimens. Perineal pattern and EP/st ratios were made using a Zeiss III compound microscope with Nomarski differential interference contrast at powers up to 1,000x magnification.

For molecular analyses, DNA of one female nematode of each RKN population was extracted and PCR assays were conducted as described by Castillo et al. (2003). The *coxII-*16S rRNA mtDNA was amplified using primers C2F3 (5′-GGTCAATGTTCAGAAATTTGTGG-3′) (Powers and Harris, 1993) and MRH106 (5′-AATTTCTAAAGACTTTTCTTAGT-3′) (Stanton et al., 1997). The reference *M*. *arenaria*, *M*. *incognita , M*. *javanica* and *M. hapla* Chitwood, 1949 populations from olive trees (*Olea europaea* L.) (Archidona-Yuste et al., 2018), and *M. hispanica* from grapevine (*Vitis vinifera* L.) (Castillo et al., 2009) previously identified and maintained on tomato in the greenhouse served as a positive control throughout this study. Detailed protocols of RFLP-PCR amplification for mtDNA fragment were studied as described previously by Powers and Harris (1993) and Maleita et al. (2012). *Hinf*I and *Dra*III digestion of amplified products was conducted using 10 μl of PCR product, 1.2 μl of the restriction enzyme buffer (10X) and 15 units of the restriction enzyme (TaKaRa Biotech). Digestion was allowed to proceed for 3 hr at 37 °C. Restricted PCR products were separated on a 2% agarose gel. This pattern was compared with Powers and Harris (1993) and Maleita et al. (2012) and our positive controls. Amplification products with approximately 650 bp were identified as *M. hapla,* on the other hand, products with approximately 1,800 bp were digested with *Hinf*I, this digestion generated two fragments of approximately 1,200 bp and 400 bp for *M. incognita* and finally, *Dra*III could not digest *M. hispanica,* but generated two fragments of approximately 1,000 and 800 pp for *M. javanica*.

Cretan mountain tea or Malotira (*Sideritis syriaca* L.) is an aromatic -medical perennial plant of the family Lamiaceae which is indigenous in the Island of Crete, naturally growing in rocky mountain areas, usually from 1,300 to 2,000 m above sea level and being recently cultivated in several areas of the island. A few plants indicating the symptoms of chlorosis and dryness with galls and egg masses in roots were found in a commercial crop in Heraklion Province, Crete, in March 2017. The crop was established in an area that had not been cultivated before and had been cleaned by natural vegetation, before planting Malotira and other aromatic-medical plants. The seed for the Malotira crop came from cultivated plants but the initial material for starting the first cultivation, had been collected from native plants of Crete, a couple of years ago. In August 2017, some more plants from the same area were uprooted and checked for nematode infection. The roots had small galls, typical of RKN infection (8–30 galls per gram of root) while in the surrounding soil were found juveniles of *Meloidogyne* at a density of 1.6 per gram of soil. *Meloidogyne hapla* was detected in the sample examined in March 2017. The species had been previously reported in Greece (Hirschmann et al., 1966; Koliopanos, 1980; Vovlas and Antoniou, 1987; Vlachopoulos, 1994) and Crete (Pyrowolakis, 1980) with identification based only on morphological characteristics. Herein, this study is the first report of molecular characterization of *M. hapla* in Greece. In other sample collected in August 2017, *M. javanica* was also detected. To our knowledge, Malotira is reported for the first time as the host of RKN worldwide. Thus, we could hypothesize that the nematode species are not indigenous in the area but introduced in the field through infested soil or plant material.

*Aloe vera* L. is a perennial plant and its cultivation has been recently expanded in Greece, mainly in lands which had been abandoned from cultivation or/and in areas with natural vegetation. Roots from cultivated aloe plants from the Island of Andros, Cyclades, with symptoms of stunting and leaf discoloration, were sent to the lab for nematode diagnosis. Longitudinal root sections were examined under a stereoscope and revealed the presence of egg masses and females of *Meloidogyne* deeply embedded inside the root tissue. The nematode species detected was identified as *M. hispanica* and it is hypothesized that the nematode was transferred through rooted propagating material. Aloe infection by *M. javanica* and *M. incognita* has been reported in Crete, Greece (Palomares-Rius et al., 2015). To our knowledge, this work consists the first report of infection of aloe by *M. hispanica* in Europe.

Corn and soybean (*Glycine max* (L) Merr) are common crops in the area of Ag. Athanasios, Drama, North Greece. Roots with surrounding soil of young corn plants (hybrid P1921) from two fields and soybean plants (variety 92B63) from one field indicating symptoms of severe stunting were sent to the lab for nematode diagnosis. The roots of corn had few galls with immature females of *Meloidogyne*. However, soybean roots were severely galled and egg masses were isolated. *Meloidogyne hispanica* and *M. incognita* were found in the two fields with corn while *M. javanica* was found in the field with soybean. *Meloidogyne hispanica* is indigenous in the area, as was also previously found in sunflower (Tzortzakakis et al., 2014). The nematode has a wide host range including corn as has been demonstrated in pot tests (Maleita et al., 2012). To our knowledge, this work consists the first report of natural infection of corn by *M. hispanica* in Europe and the first report of infections of corn by *M. incognita* and soybean by *M. javanica* in Greece.
